# The Business Management of the Independent Practitioner and the N. Y. Universal Purchasing Co.

**Published:** 1881-06

**Authors:** 


THE BUSINESS MANAGEMENT OF THE INDEPENDENT PRACTITIONER AND THE N.Y. UNIVERSAL PURCHASING COMPANY.—Up to this issue of the journal the proprietor has been under a heavy strain—mental and physical—with his professional duties, editorial work and looking after a careless set of printers; therefore the business management and correspondence was necessarily, to a great extent, turned over to clerks. The result has given dissatisfaction to some. We have made errors, but not intending injustice to anyone, and we regret that a few have misinterpreted our free-spoken and open business method. We have made a decided change in our business plans, which we hope will be acceptable to all our friends and relief to ourself. We have learned that it requires ability, good business management, energy, patience, and a large sum of money to succeed with a medical journal. In all these we have endeavored to come up to the requirements, but are not fully satisfied with all our efforts. We shall endeavor to make our dearly-bought experience redound to the profit of our friends as well as ourselves.

Through the New York Universal Purchasing Company we have arranged to have our printing and business management of the journal done in New York City by Capt. Wm. Miller, who is also manager of the N. Y. U. P. Co. We have known Capt. Miller personally for thirteen years, and found him to be a thorough gentleman, a most excellent business man, with unusual knowledge of all kinds of merchandise. His business management of THE INDEPENDENT PRACTITIONER will tell its friends of his worth and ability, and we ask all to aid us in our renewed energy and *new home.* We propose after this to have the journal mailed on the *first* of each month, and we hope all who have not already done so will remit promptly, cheering us on to success in our efforts to uphold right, truth and justice, which shall be rendered to everyone with boldness and independence.

The N. Y. Purchasing Co. is a newly-organized agency, and can not be too highly commended to those of our friends who wish to buy goods, wholesale or retail, in New York City. As many of our dental friends expect to attend the dental meetings next August in New York City, we make, for them and others, the following statement: Those desiring to visit New York can save time and money, at any season of the year, by corresponding or calling on this company, which will give information about hotels, boarding-houses, railroad, ocean and river steamboat tickets, general merchandise, etc.

